# Francis Thompson: Poet and Medical Student

**Published:** 1909-12-25

**Authors:** 


### FRANCIS THOMPSON:

### POET AND MEDICAL STUDENT.

Poetry and medicine have not gone hand in hand so often that we can afford to pass by modern examples of their union. To-day, too, when the profession of law, like some deep-sea octopus, aspires to gather within its tentacles the prizes of all the professions, we can congratulate ourselves that if law has gained much in the province of politics, medicine has gained more in the kingdom of poetry. There are few lawyers among the poets. That is probably because the genius of law is its conservatism, and the genius of poetry its innovation, a childish freshness of perception, which only by seeing with new eyes and hearing with new ears can keep itself alive. Medicine, however, lies between these two extremes; there is room in it for the inductive methods of the lawyer, and for the deductive mind of the poet. In the last century Keats was poet and apothecary; at the present moment Mr. Robert Bridges is poet as well as M.D. Medicine has her right of place among the muses.

It is not often, however, that medicine can claim her share in a poet absolutely of the first rank. Yet in Francis Thompson, Keats has gained an eternal contemporary, whose life too was saddened by disease and poverty, and, if not cut short in early manhood, hardly carried him into his prime. In 1859—the son of a doctor we may note—Francis Thompson was born at Preston, and two years ago, on November 13, 1907, he died in London at the zenith of his fame. His life—it may be well to mention a few facts about it, since it is death alone which distinguishes the eminent from the successful—was characteristically the life of a poet. There is a common story for poets and artists, which the circumstance of varying centuries alters but little; they seem to be the beloved of Fortune, if we may judge by the variety of gifts, the surprising changes of attention she bestows. Francis Thompson was educated at Ushaw College, near Durham, and being destined to succeed his father in the profession of medicine, was sent as a young man to Owens College, Manchester. Then the inevitable happened. The apparently innocent young fellow, whose feet seemed firmly planted on the normal pathway of life, proved—

One stricken from his birth
With curse
Of destinate verse.

Medicine, as a profession, was abandoned, and he passed through an emotional crisis, which meant a breach with his family and left him stranded on the rocks of disease. He left Manchester for London, and found her streets, as many another poet has done, the *via dolorosa* to fame. He gained the bare necessities of life as his wits allowed him, working in various small shops, being always headed off towards the Embankment, which, like a live thing, seemed to mark him as its prey. In a wonderful passage he tells the story to a child friend of his later and happier years:—

Once, bright Sylviola! in days not far,
Once—in that nightmare time which still doth haunt
My dreams, a grim, unbidden visitant—
Forlorn, and faint, and stark,
I had endured through watches of the dark
The abashless inquisition of each star,
Yea, was the outcast mark
Of all those heavenly passers' scrutiny;
Stood bound and helplessly
For Time to shoot his barbèd minutes at me;
Suffered the trampling hoof of every hour
In night's slow-wheelèd car;
Until the tardy dawn dragged me at length
From under those dread wheels; and, bled of strength,
I waited the inevitable last.
Then there came past
A child; like thee, a spring-flower; but a flower
Fallen from the budded coronal of Spring,
And through the city streets blown withering.
She passed—O brave, sad, lovingest, tender thing!—
And of her own scant pittance did she give,
That I might eat and live:
Then fled, a swift and trackless fugitive.
. . . . . . . . .

And ah! so long myself had strayed afar
From child, and woman, and the boon earth's green,
And all wherewith life's face is fair beseen;
Journeying its journey bare
Five suns, except of the all-kissing sun
Unkissed of one;
Almost I had forgot
The healing harms,
And whitest witchery, alurk in that
Authentic cestus of two girdling arms.

Rarely has the Embankment found so splendid a music, and rarely has any poet so exquisitely caught the beauty of childhood as Francis Thompson, who found in it the healing touch for the scars with which life had marked him. Some magazine verses attracted the notice of some Roman Catholics, who found him out, gave him the medical treatment which he needed, and with whom he spent the remainder of his life. Storrington, in Sussex, was where his broken spirit healed itself and gave out, perhaps because of its bruises, the sweetness of crushed leaves in his songs.

His genius quickly expanded, and was, doubtless because of its extraordinary individuality and overwhelming claims, quickly recognised and championed. Coventry Patmore's praise disarmed the arrows of adverse criticism, and it is Patmore's influence, together with that of Spenser, Crashaw, and Shelley, which is most strongly to be found in his work. His finest piece of sustained writing is, of course, "The Hound of Heaven," in which poem all his qualities are revealed: his amazing Oriental exuberance of language, his religious mysticism, his fecundity of imagery, his mastery of metrical effect, his rich mine of language. It is a masterpiece, a poem indeed with only one defect—a foolish oxymoron of four lines in the second stanza. "The Hound of Heaven" is an ode which writes the romance of the soul, of the soul of the mystic at least which, touched with the untempered and restless curiosity of the modern mind, searches after beauty in all the byways of the world—in philosophy, in the laughter and tears of ordinary life, in love of friends and relations,

> By many a hearted casement, curtained red,
> Trellised with intertwining charities, in childhood, which had a unique appeal to him, in nature, and at last, after many wanderings and disappointed hopes, finds in God, as the Catholic Church has defined him, the satisfaction and peace which he had failed to win elsewhere. It will be seen that this ode is great, not because it gives us a new teaching—Thompson had no message in that sense—but because out of the familiar symbolism of the Church a poet was able to create a new beauty, to relight the lamp of truth which every symbolism contains, and which without symbols cannot be expressed at all. To a mind which delights in the energies rather than in the subleties of beauty, the luxuriance of the Church's symbolism must inevitably appeal, and perhaps the chief relic which the West has preserved of the East is the gorgeous raiment and magnificent ceremonial of the Bride of Christ in the Church's ritual. It was this side of the Church's mysticism, not the subtler, more intellectual mysticism of Patmore or Traherne, which invested Francis Thompson's temperament, and this accounts for the influence of Crashaw's poetry upon him. We can well imagine the effect of the lines on "The Book and Picture of the Seraphical S. Teresa" upon Thompson. They must have awakened many a latent chord, for poetry plays to the soul the same mysterious part as the moon plays to the tide-waves of the sea, directing from her own high throne the spirit's ebb and flow. It was the imaginative mysticism, then, rather than the subtle intellectual mysticism to which his genius belonged. We may be thankful that it was so, as the Crashaws of the world will always be greater poets than the Donnes of it. It is better to be intoxicated with words than with dialectic, and Thompson was certainly intoxicated with words.

But his religious mysticism did not absorb his whole energies, and he retained his liking for science to the end. As to all lovers of words, technical vocabularies had a fascination for him.

Writing of Shelley, he says: "The dimmest sparked chip of a conception blazes and scintillates in the oxygen of his mind"; and once more: "Where our dull eyes see only ruin, the finer eye of science has discovered life in putridity and vigour in decay, seeing dissolution even and disintegration, which in the mouth of man symbolise disorder, to be in the works of God undeviating order, and the manner of our corruption to be no less wonderful than the manner of our health." Indeed, his whole prose essay, "Health and Holiness," is a plea, based on scientific grounds, for the rights of the body against an undue asceticism; it shows that the body has its revenges for those who are scornful of its needs, illustrating from history that men and women to-day are of very different vigour, if of finer nervous quality, than, say, even the men of Elizabethan England. It is curious to find a mystic asking scientific questions of St. Francis, St. Ignatius, and by implication St. Teresa. It is a tract written by the man of letters for the man of science, on psychotherapy.

The prose of poets is always fascinating; but the prose of Francis Thompson would require an essay to itself. His treatment of childhood in prose and verse alike, his power of giving the essence of its charm to those who are no longer children is unique. There is an astonishing passage in his "Sister Songs" on the sex which is latent in children. The poem is really a marvellous array of metaphors descriptive of this, and the present writer knows no finer example of crystallising into a metaphor the peculiar assurance of protection which innocence gives to all young things than his:

> The dragon to its own Hesperides.

It is indeed such lines as these for which his poetry is unique. With a few examples this fragment of a note on him must end. For the startling effect of light:

> The butterfly sunset claps its wings.

For the magic of colour:

> Mark yonder, how the long laburnum drips
> Its jocund spilth of fire, its honey of wild flame.

A reminiscence of Tennyson; but Thompson always justified his takings by improving the original.

For the exuberant joy of spring:

> Let even the slug-abed snail upon the thorn
> Put forth a conscious horn!

For the limitless bounds of the imagination:

> And all man's Babylons strive but to impart
> The grandeurs of his Babylonian heart.

But he is a poet impossible almost to quote from. One remembers poems and passages rather than lines. One more example must suffice. In asking, as all poets have done, for fit words to express his vision, he cries:

Or if that language yet with us abode
Which Adam in the garden talked with God!

His poems are encrusted with such metaphors, and the mystic, the scientist, the scholar each had his part in them. These four (for the mystic includes both the artist and the saint) have each had his own symbols for the first principle. The artist calls it Beauty, and not caring for definitions, attempts to create it. The saint calls it God, and worships it. The scientist calls it the ultimate energy, and tries to discover it. The scholar calls it Truth, and is always trying to define it. When either of these has tried to define the aim and practice of the other—mysticism, art, religion, philosophy—he has always gone astray. But were the scientist, after reading Francis Thompson, again to attempt the impossible, he might say that art is the energy of beauty at the degree thermal for its flash-point. It was at least the energy of beauty which produced the incomparable splendours of this poetry, and which gave to it the lurid lights and the black shadows of a city on fire. Being a poet for poets and amateurs of letters, he has not yet been fully recognised outside that elect circle, and it is in the hope of adding to his circle of admirers that this note on him appears, containing what should be, and will be in many instances, known already to medical men.

